# No evidence for protective erythropoietin alpha signalling in rat hepatocytes

**DOI:** 10.1186/1471-230X-9-26

**Published:** 2009-04-21

**Authors:** Thorsten Bramey, Patricia Freitag, Joachim Fandrey, Ursula Rauen, Katja Pamp, Jochen Erhard, Stilla Frede, Herbert de Groot, Frank Petrat

**Affiliations:** 1 Institut für Physiologische Chemie, Universitätsklinikum Essen, Hufelandstrasse 55, D-45122 Essen, Germany; 2 Institut für Physiologie, Universitätsklinikum Essen, Hufelandstrasse 55, D-45122 Essen, Germany; 3 Klinik für Chirurgie/Viszeral- und Gefäßchirurgie, Evangelisches Krankenhaus Dinslaken, Kreuzstraße 28, 46535 Dinslaken, Germany

## Abstract

**Background:**

Recombinant human erythropoietin alpha (rHu-EPO) has been reported to protect the liver of rats and mice from ischemia-reperfusion injury. However, direct protective effects of rHu-EPO on hepatocytes and the responsible signalling pathways have not yet been described. The aim of the present work was to study the protective effect of rHu-EPO on warm hypoxia-reoxygenation and cold-induced injury to hepatocytes and the rHu-EPO-dependent signalling involved.

**Methods:**

Loss of viability of isolated rat hepatocytes subjected to hypoxia/reoxygenation or incubated at 4°C followed by rewarming was determined from released lactate dehydrogenase activity in the absence and presence of rHu-EPO (0.2–100 U/ml). Apoptotic nuclear morphology was assessed by fluorescence microscopy using the nuclear fluorophores H33342 and propidium iodide. Erythropoietin receptor (EPOR), EPO and Bcl-2 mRNAs were quantified by real time PCR. Activation of JAK-2, STAT-3 and STAT-5 in hepatocytes and rat livers perfused in situ was assessed by Western blotting.

**Results:**

In contrast to previous in vivo studies on ischemia-reperfusion injury to the liver, rHu-EPO was without any protective effect on hypoxic injury, hypoxia-reoxygenation injury and cold-induced apoptosis to isolated cultured rat hepatocytes. EPOR mRNA was identified in these cells but specific detection of the EPO receptor protein was not possible due to the lack of antibody specificity. Both, in the cultured rat hepatocytes (10 U/ml for 15 minutes) and in the rat liver perfused in situ with rHu-EPO (8.9 U/ml for 15 minutes) no evidence for EPO-dependent signalling was found as indicated by missing effects of rHu-EPO on phosphorylation of JAK-2, STAT-3 and STAT-5 and on the induction of Bcl-2 mRNA.

**Conclusion:**

Together, these results indicate the absence of any protective EPO signalling in rat hepatocytes. This implies that the protection provided by rHu-EPO in vivo against ischemia-reperfusion and other causes of liver injury is most likely indirect and does not result from a direct effect on hepatocytes.

## Background

There is increasing evidence that erythropoietin alpha (EPO) has cell- and tissue-protective properties independent of its hematopoietic activity [1-3]. In the liver, treatment of rats and mice with recombinant human erythropoietin alpha (rHu-EPO) significantly reduced ischemia-reperfusion injury as indicated by decreases in histopathological scores, release of liver enzymes, markers of apoptosis, injurious intracellular signalling, and reactive oxygen species formation [4-9]. In line with these results rHu-EPO also promoted hepatic regeneration after partial liver resection [10] and attenuated liver injury associated with hemorrhagic shock [11].

The signalling pathways of rHu-EPO in non-erythroid cells which are responsible for the protection provided by EPO are still under investigation and are not yet completely resolved. As for erythroid progenitors the presence and functionality of the EPO receptor (EPOR), a member of the type I cytokine receptor family [12,13], appears to be required. While it is known that in erythroid progenitors the EPOR exists as a preformed homodimer, which binds EPO, it has been suggested that in non-hematopoietic tissues a heterodimer of the EPOR and the common β-chain of the interleukin-3 receptor may mediate anti-apoptotic effects of EPO [14]. Classical EPOR activation depends on the recruitment of Janus tyrosine kinase 2 (JAK-2) and subsequent activation of signal transducer and activator of transcription 5 (STAT-5) [15]. However, a variety of additional signal transduction pathways have been proposed. In this context, signalling via signal transducer and activator of transcription 3 (STAT-3) is considered to be involved in transducing EPO-dependent cell protection in non-erythroid tissues [16-18]. B-cell lymphoma 2 (Bcl-2) is among the target genes that confer the anti-apoptotic action of EPO in hematopoietic progenitor cells [19].

Here we report the unexpected finding that rHu-EPO did not protect primary cultured rat hepatocytes against hypoxic injury, hypoxia-reoxygenation injury and against cold-induced, free radical-mediated apoptosis. Subsequent studies with cultured hepatocytes and the rat liver perfused in situ strongly indicate the absence of EPO signalling in rat hepatocytes to account for the missing protection by the cytokine.

## Methods

### Materials

Recombinant human erythropoietin alpha (rHu-EPO; stocks: commercial ERYPO^® ^Product Line, 10,000 or 40,000 U/ml; 5.0 mg glycine/ml, preservative free without albumin and citrate) was kindly provided by Janssen-Cilag (Neuss, Germany). Primers for real-time polymerase chain reactions (PCRs) were obtained from Invitrogen (Karlsruhe, Germany), the RC DC Protein Assay^® ^was purchased from Bio-Rad (Munich, Germany), and avian myeloblastosis virus reverse transcriptase from Promega (Heidelberg, Germany). Fetal calf serum, 2,2'-dipyridyl, Leibovitz L-15 medium, and Ponceau S came from Sigma-Aldrich (Taufkirchen, Germany). The fluorescent dye SYBR Green^® ^(for real time PCR) was obtained from Eurogentec (Verviers, Belgium). The Primer Express software and the Gene Amp 5700 Sequence Detection System were obtained from Applied Biosystems (Weiterstadt, Germany). For the determination of total and phosphorylated STAT-3 and STAT- 5 and phosphorylated JAK-2 the PhosphoPlus^®^Stat3 (Tyr705), the PhosphoPlus^®^Stat5 (Tyr694) and the Phospho-Jak2 (Tyr1007/1008) antibody kits from Cell Signalling Technology^® ^(Frankfurt, Germany) were used, respectively. The NE-PER^® ^nuclear and cytoplasmic extraction kit was purchased from Pierce Biotechnology (Schwerte, Germany), EPOR antibodies C-20 (sc695) were from Santa Cruz (Heidelberg, Germany) and gas mixtures from Messer Griesheim (Krefeld, Germany).

### Animals

Male Wistar rats (300–350 g) were obtained from the Zentrales Tierlaboratorium (Universitätsklinikum Essen). Animals were kept under standard conditions with free access to food and water. All animals received humane care in compliance with the institutional guidelines.

### Perfusion of the rat liver in situ

Rat livers were perfused as previously described [20] with some modifications. Briefly, subsequent to ketamine/xylazine anaesthesia (80 mg and 6 mg, respectively, per kg body weight via intraperitoneal injection) animals underwent median laparotomy. Then the portal vein was cannulated and a peristaltic pump used to start perfusion of the liver at 30 ml/min with warm (37°) modified Krebs-Henseleit (KH) buffer (115 mM NaCl, 25 mM NaHCO_3_, 5.9 mM KCl, 1.2 mM MgCl_2_, 1.2 mM NaH_2_PO_4_, 1.2 mM Na_2_SO_4_, 2.5 mM CaCl_2_, 20 mM HEPES, pH 7.4) that had been equilibrated with 95% O_2_/5% CO_2 _(carbogen); to avoid congestion of the liver, the *Vena cava inferior *was cut distally from the *Venae renalis *immediately after perfusion had started. Subsequent to thoracotomy the perfusate was drained through a second cannula that had been placed within the suprahepatic *Vena cava inferior *via the right atrium and the infrahepatic *Vena cava inferior *was ligated between the liver and the *Venae renalis *to avoid loss of the perfusate. After a short (2–3 minutes) perfusion period that was required to completely remove the blood within the liver, rHu-EPO was added to the reservoir bottle and rapidly mixed with the KH buffer to gain 8.9 U/ml; in controls no rHu-EPO was added. Then non-recirculating in situ liver perfusion was continued for further 15 minutes. Afterwards, the complete liver was removed and rapidly frozen in liquid nitrogen until tissue processing for analytic assays.

### Isolation of the hepatocytes, culture and pre-treatment with rHu-EPO

Hepatocytes were isolated from male Wistar rats, seeded onto collagen-coated culture flasks or glass coverslips and cultured as described previously [21]. Experiments were started 20–22 hours after isolation of the cells. Hepatocytes were pre-incubated with rHu-EPO (0.2–100 U/ml) for 15 minutes, 2, 3, 18 or 20 hours (over night, subsequent to their isolation) in L-15 medium at 74% N_2_/21% O_2_/5% CO_2 _(37°C, humidified atmosphere).

### Culture of UT-7 cells with rHu-EPO

UT-7 cells, an erythropoietin-responsive hematopoietic cell line [22], which was generously provided by P. Mayeux (INSERM 152, Hopital Cochin, Paris, France), served as a positive control for EPOR signalling. UT-7 cells were grown in suspension in RPMI 1640 cell culture medium from BioWitthaker (Verviers, Belgium) supplemented with 10% fetal bovine serum from Biochrom (Berlin, Germany) and 1 U rHu-EPO/ml; cells were splitted twice/week. For experiments, cells were used at a density of 10^6 ^cells/ml and kept in medium without rHu-EPO for 1 hour before the start of the experiment.

### Induction of hypoxia/reoxygenation in cultured hepatocytes

At the beginning of the experiments, hepatocytes were washed three times with Hanks' balanced salt solution (137.0 mM NaCl, 5.4 mM KCl, 1.0 mM CaCl_2_, 0.5 mM MgCl_2_, 0.4 mM KH_2_PO_4_, 0.4 mM MgSO_4_, 0.3 mM Na_2_HPO_4_, 25.0 mM HEPES, pH 7.4) and then covered with KH buffer at 37°C. Normoxic incubations (controls) were performed in a humidified atmosphere of 74% N_2_/21% O_2_/5% CO_2_. Hypoxic conditions were established by saturating the incubation solution with 95% N_2_/5% CO_2 _before adding it to the cells, followed by gentle flushing of the culture flasks with the gas mixture through cannulae piercing the rubber stoppers of the flasks, as described previously [21,23,24]. After locking the flasks, cells were incubated for 5–6.5 hours within an incubator at 37°C. The flasks were again flushed with the respective gas mixtures each time a sample was taken. RHu-EPO (0.2–100 U/ml) was added to the KH buffer just prior to its addition to the cells. Some experiments were performed with rHu-EPO that had been heat-inactivated by boiling for 30 minutes. Where indicated, glycine (66 μM, 0.66 mM or 10 mM) was added to the cells prior to the start of the hypoxic treatment. Reoxygenation of the cells was performed after different hypoxic periods by gassing the flasks with 74% N_2_/21% O_2_/5% CO_2 _for 2 minutes followed by an incubation in this atmosphere within an incubator at 37°C. Moderate hypoxia was achieved by placing the culture dishes in an incubator with 1% or 3% O_2 _(5% CO_2 _and N_2 _as balance) for the indicated time periods.

For fluorescence microscopy, 6-well cell culture plates, containing collagen-coated coverslips with adherent hepatocytes that had been pre-treated or not with rHu-EPO for 20 hours, washed and supplied with KH buffer ± rHu-EPO (10 or 100 U/ml), were placed in air-tight vessels that were then flushed for 10 minutes either with 74% N_2_/21% O_2_/5% CO_2 _or 95% N_2_/5% CO_2_. After locking the vessels, cells were incubated for 2–4 hours at 37°C; note that hepatocytes died more slowly in the hypoxic vessel than in culture flasks (see above), as pre-equilibration of the medium with 95% N_2_/5% CO_2 _was not possible. Afterwards, the buffer was removed and the cells were incubated for 24 hours in L-15 medium (37°C) again with or without rHu-EPO at 74% N_2_/21% O_2_/5% CO_2_.

### Induction of cold-induced apoptosis

Hypothermic injury was induced according to refs. [25,26]. Cultured hepatocytes were washed with Hanks' balanced salt solution and cells covered with University of Wisconsin solution or L-15 medium with or without rHu-EPO (2, 10, 50 or 100 U/ml; medium supplemented as described previously [26]) at room temperature. Incubations in cell culture medium were performed in air-tight vessels which were flushed with 74% N_2_/21% O_2_/5% CO_2_, cells in University of Wisconsin solution were exposed to room air; cells were incubated at 4°C for 24 hours. In some experiments, the iron chelator 2,2'-dipyridyl (100 μM) was added at the beginning of the cold incubation as a positive control for protection from cold-induced injury in University of Wisconsin solution and, in other experiments, in order to assess an effect of rHu-EPO to the weaker iron-independent component of cold-induced apoptosis to hepatocytes in L-15 medium [27,28]. Increases in the hepatocyte chelatable iron pool were provoked in cell culture medium at 74% N_2_/21% O_2_/5% CO_2 _by the addition of the membrane-permeable Fe(III)/8-hydroxyquinoline complex ([29]; prepared as described previously [30]).

### Assessment of cellular and nuclear alterations (apoptotic vs. necrotic cell death)

Hepatocyte morphology after various periods of hypoxia and reoxygenation, respectively, was assessed by phase contrast microscopy. Nuclear morphology was assessed by fluorescence microscopy according to ref. [26]. Twenty fields of vision (original magnification: × 400) à 15–30 hepatocytes were visually evaluated (blinded) per condition and experiment.

Loss of cell viability was assessed by the determination of extracellular, i.e. released, lactate dehydrogenase (LDH) activity; released LDH activity is given as percentage of total LDH activity [26].

### Detection and quantification of EPOR, EPO and Bcl-2 mRNA

Isolation of total RNA from cultured hepatocytes and perfused liver, reverse transcription into cDNA and real-time PCR were performed as described [31]. Primers for real-time PCR were designed to yield amplicon sizes of 150 bp, annealing temperature of 60°C and CG content of about 60%. Primers for real-time PCRs were: EPOR upstream EPOR1 5'-*ccg gga tgg gct tca act ac*-3' and downstream EPOR2 5'-*tcc agt ggc aca aaa ctc gac-*3' spanning nucleotides 291 – 441 or for detection of full length EPOR mRNA EPOR3 5'-*ggg cta cat cat gga cca act c-3'*and EPOR4 5'-*ggc tgg agt cct agg agc agg cc*-3' spanning nucleotides 71 – 1616 of rat EPO receptor sequence NM_017002.2. Primers for EPO mRNA were upstream 5'-*ggt cac ctg tcc cct ctc ct *-3' and downstream 5'-*ctg gag tgt cca tgg gac ag*-3' and for Bcl-2 upstream *5*'-*gga cgc gaa gtg cta ttg g-3*' and downstream *5*'-*ccg aac tca aag aag gcc ac-3*', respectively. The identities of the amplification products for EPOR were verified by sequencing (SEQLAB; Göttingen, Germany).

### Determination of activated JAK-2, STAT-3 and STAT-5

Cultured rat hepatocytes and UT-7 cells were treated with 10 U rHu-EPO/ml for 15 minutes and rat livers perfused in situ with 8.9 U/ml rHu-EPO (for 15 minutes). Cellular extracts were prepared using the NE-PER^® ^nuclear and cytoplasmic extraction kit and used for Western blotting to detect nuclear phosphorylated and thus activated JAK-2 (pJAK-2), STAT-3 (pSTAT-3) and STAT-5 (pSTAT-5) exactly as described in the protocol from Cell Signalling Technology^®^. As positive controls, extracts from the EPO-responsive cell line UT-7 were used. Western blotting and detection were performed as described in Frede et al. [32].

### Statistics

All experiments were performed in duplicate and repeated at least 3 times. Traces and blots shown in the figures are representative for all the corresponding experiments carried out. Data are expressed as mean values ± or + SD. Data obtained from two groups were compared by means of Student's *t *test (matched values, two-tailed, paired) and comparisons among multiple groups were performed using an analysis of variance (ANOVA). A *p*-value of < 0.05 was considered significant.

## Results

### Effect of rHu-EPO on hypoxia-reoxygenation injury to cultured rat hepatocytes

For the induction of hepatocellular injury, occurring either already during the hypoxic period or upon reoxygenation oxygen partial pressures (pO_2_) below 0.3 mm Hg, i.e. deep hypoxia (anoxia), is required [33]. The mechanism of injury in the hypoxic period (like the mechanism of hepatocellular injury in the ischemic liver) is considered to be necrotic [34,35]. Upon reoxygenation, the mode of hepatocellular injury is highly variable ranging from necrotic to apoptotic cell death (again similar to the situation in the reperfused liver [34,35]).

**Figure 1:**
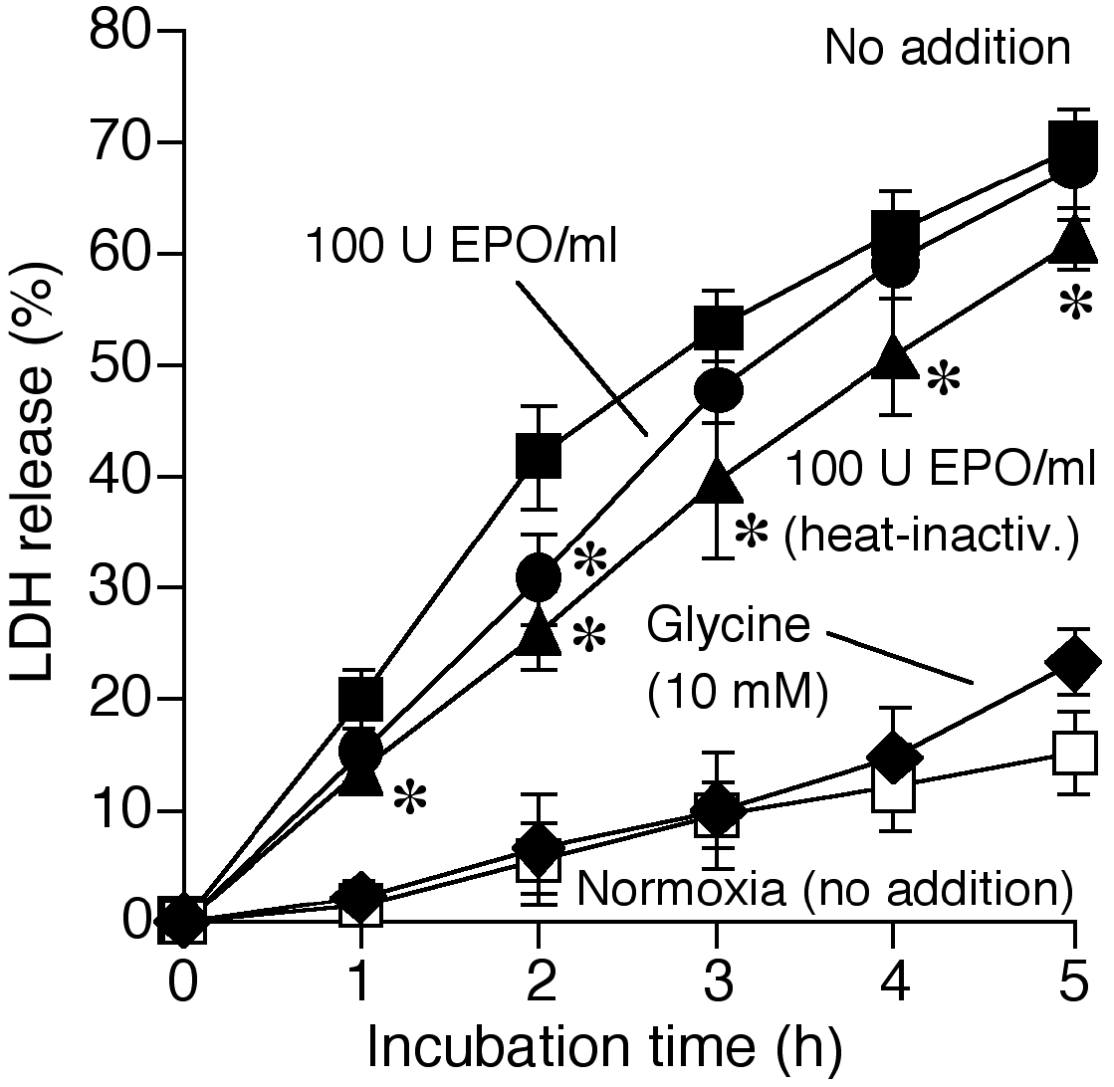
**Effect of rHu-EPO on the hypoxic injury to cultured hepatocytes**. Cultured rat hepatocytes were pre-incubated in the presence or absence of rHu-EPO (100 U/ml) in L-15 medium (37°C) for 20 hours at normoxia (74% N_2_/21% O_2_/5% CO_2_). Then the cells were incubated in modified Krebs-Henseleit (KH) buffer (37°C) for 5 hours, again with or without rHu-EPO, under deep hypoxia (gassing with 95% N_2_/5% CO_2_; closed symbols) or normoxic conditions (open symbol). In some experiments glycine (10 mM) was added before starting the hypoxic treatment; other experiments were performed using heat-inactivated (30 minutes at 100°C) rHu-EPO. Cell injury was determined by the release of cytosolic lactate dehydrogenase (LDH). Values shown represent means ± S.D. of 4 independent experiments. *p < 0.05 vs. respective incubations at hypoxia with no addition; glycine (10 mM) provided significant protection (*p *< 0.05) vs. respective incubations at hypoxia (not indicated). Closed squares: hypoxia, no addition; closed circles: hypoxia, 100 U rHu-EPO/ml; closed triangles: hypoxia, 100 U heat-inactivated rHu-EPO/ml; closed diamonds: hypoxia, 10 mM glycine; open squares: normoxia, no addition.

When cultured rat hepatocytes were incubated under hypoxic conditions, 70 ± 2% of the cells lost their viability during 5 hours of incubation (Figure 1). Glycine (10 mM) largely protected the cells, in line with previous reports [23,24,36-38]. In contrast to glycine, rHu-EPO (≥ 10 U/ml; 2 or 20 hours pre-incubation) only slightly attenuated hepatocyte hypoxic death (Figure 1; data shown for 100 U rHu-EPO/ml and 20 hours pre-incubation). Furthermore, based on experiments with heat-inactivated rHu-EPO and glycine, this effect could be fully explained by the presence of contaminant glycine (0.66 mM in samples with 100 U rHu-EPO/ml) used by the manufacturer to stabilize the rHu-EPO stock solution. Hepatocytes appeared to be protected even more effectively in experiments with heat-inactivated rHu-EPO than in experiments with the intact hormone (Figure 1), suggesting a moderate negative effect of the latter. At normoxia, however, pre-incubation of the cells with rHu-EPO (0.2–100 U/ml for up to 20 hours) in L-15 medium and incubation in KH buffer had no effect at all on cell viability (data not shown; n = 5).

**Figure 2:**
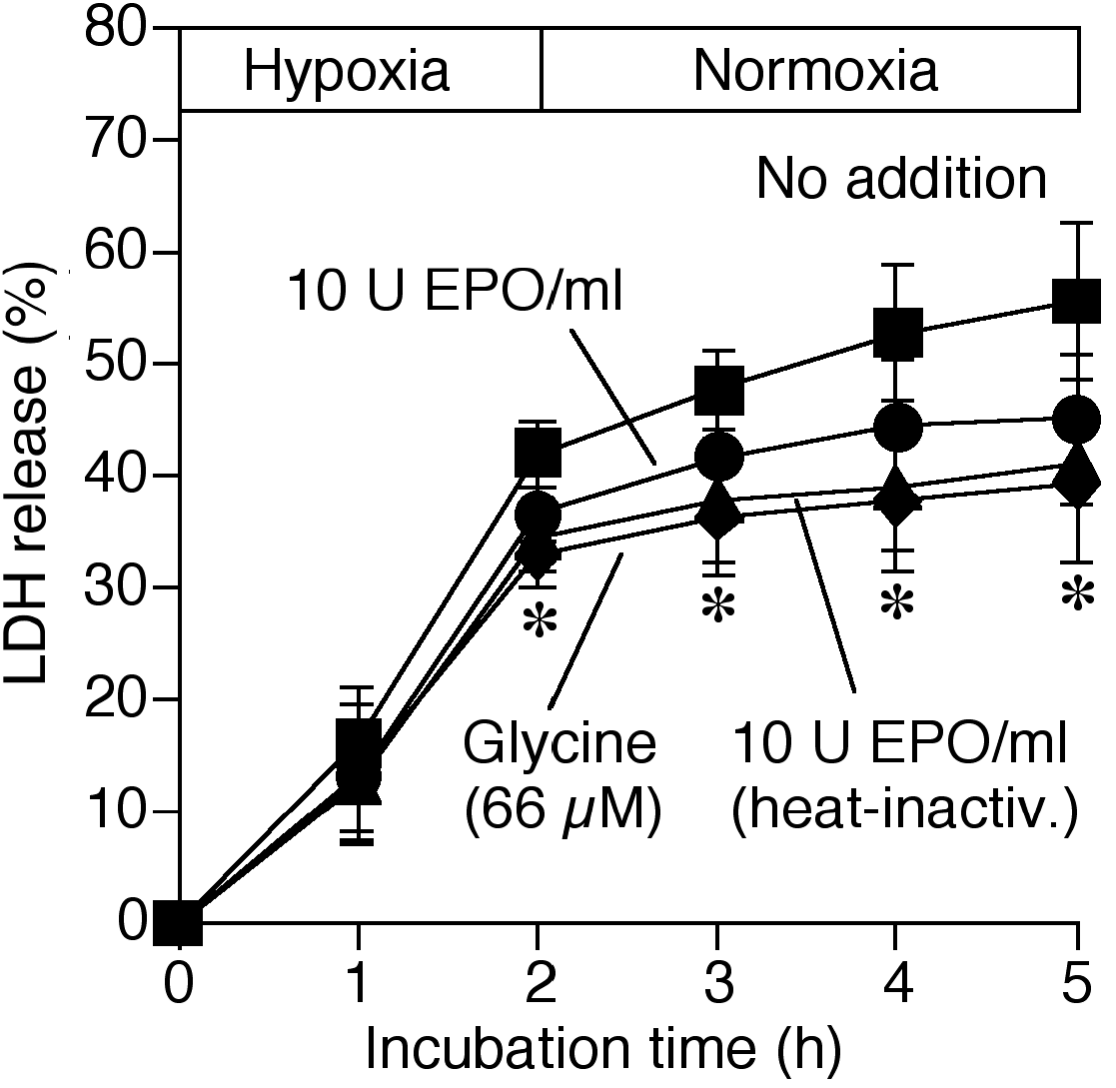
**Effect of rHu-EPO on the reoxygenation injury to cultured hepatocytes**. Cultured rat hepatocytes were pre-incubated in the presence or absence of rHu-EPO (10 U/ml) in L-15 medium (37°C) for 20 hours at normoxia (74% N_2_/21% O_2_/5% CO_2_). Then the cells were incubated in modified Krebs-Henseleit (KH) buffer, again with or without rHu-EPO, under deep hypoxia (for 120 minutes) followed by normoxia (37°C). In some experiments glycine (66 μM) was added before starting the hypoxic treatment; other experiments were performed using heat-inactivated (30 minutes at 100°C; heat-inactiv.) rHu-EPO. Cell injury was determined by the release of cytosolic lactate dehydrogenase (LDH). Values shown represent means ± S.D. of 4 independent experiments. *p < 0.05 (66 μM glycine *and *10 U EPO/ml, heat-inactiv.) vs. respective incubations at hypoxia with no addition. Closed squares: hypoxia/reoxygenation, no addition; closed circles: hypoxia/reoxygenation, 10 U rHu-EPO/ml; closed triangles: hypoxia/reoxygenation, 10 U heat-inactivated rHu-EPO/ml; closed diamonds: hypoxia/reoxygenation, 66 μM glycine.

Subsequent to 30 or 60 minutes of hypoxia (not shown; n = 4 each), reoxygenation only slightly increased the loss of cell viability. Reoxygenation injury became more obvious when the hypoxic period was prolonged to 2 hours (Figure 2). As in experiments with permanent hypoxia, rHu-EPO (≥ 10 U/ml; 2 or 20 hours pre-incubation) provided moderate protection (data shown for 10 U rHu-EPO/ml and 20 hours pre-incubation). Again, this protective effect was most likely mediated by glycine, as disclosed in controls with the amino acid and the heat-inactivated hormone, respectively.

**Figure 3:**
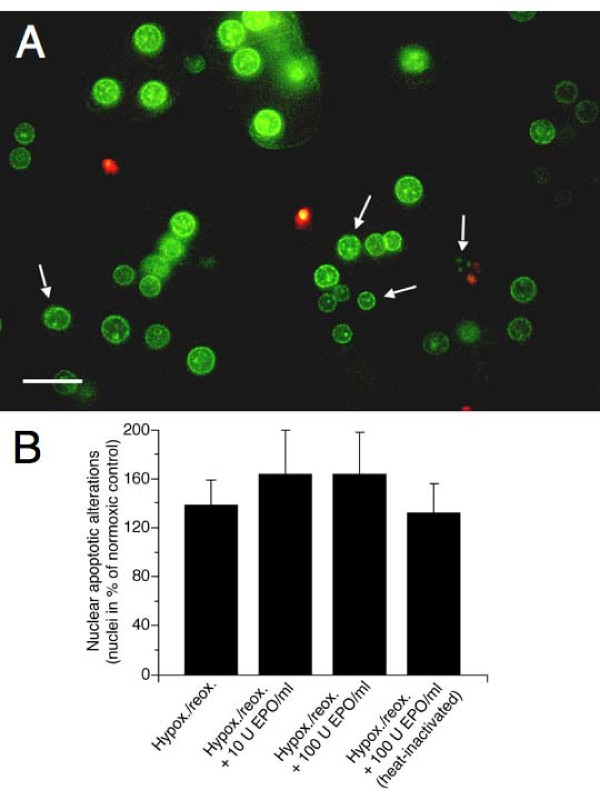
**Effect of rHu-EPO on the changes of nuclear morphology of cultured hepatocytes as induced by hypoxia/reoxygenation**. Cultured rat hepatocytes on collagen-coated glass coverslips in 6-well cell culture plates were pre-treated or not with rHu-EPO (10 or 100 U/ml) in L-15 medium (37°C) for 20 hours. Then the cells were supplied with KH buffer with or without rHu-EPO (10 or 100 U/ml) and placed in air-tight vessels that were flushed for 10 minutes either with 74% N_2_/21% O_2_/5% CO_2 _or 95% N_2_/5% CO_2_. Cells were then incubated for 3 hours at 37°C. Afterwards, the buffer was removed and the cells were incubated for 24 hours in L-15 medium (37°C) again with or without rHu-EPO at 74% N_2_/21% O_2_/5% CO_2_. Nuclear morphology was assessed by fluorescence microscopy (λ_exc _= 365 ± 12.5 nm, λ_em _≥ 515 nm; original magnification × 400) after double-staining of the cells with the membrane-permeable DNA-binding fluorochrome H33342 (1 μg/ml; green fluorescence) and the DNA-binding fluorochrome propidium iodide (5 μg/ml), that is impermeable to the intact plasma membrane but stains nuclei of necrotic and late apoptotic cells (red fluorescence). Twenty fields of vision à 15–30 hepatocytes were visually evaluated (blinded) per experiment. In (A) the effect of hypoxia/reoxygenation on the nuclear morphology is shown; bar represents 20 μm. (B) shows the effect of rHu-EPO on nuclear apoptotic alterations occurring after hypoxia/reoxygenation (hypox./reox.) in % of the normoxic controls. The microfluorographs shown are representative for three experiments with hepatocytes from different animals; bars represent means + S.D. of 3 independent experiments. Nuclear apoptotic alterations were defined as nuclear condensation, ruffling and/or fragmentation (white arrows) that had already occurred in cells that did not take up propidium iodide.

Since the onset of cell injury induced by hypoxia and developing during reoxygenation may be a late event, the effect of rHu-EPO on reoxygenation injury was studied 24 hours after a sublethal hypoxic period (Figure 3). Double staining with the nuclear fluorophores H33342 and propidium iodide revealed that under these conditions reoxygenation predominantly resulted in hepatocyte apoptotic alterations. Nuclei were condensed and/or ruffled and condensation of chromatin was detectable in cells that still excluded propidium iodide. However, chromatin margination and nuclear fragmentation was hardly observed. The cells with primarily apoptotic nuclei finally took up propidium iodide, suggesting the occurrence of secondary necrosis. RHu-EPO (10 and 100 U/ml; 20 hours pre-incubation and for 24 hours after hypoxia) did not diminish these apoptotic alterations but even slightly enhanced them (not significantly) as compared with the heat-inactivated protein, which had no effect.

### Effect of rHu-EPO on cold-induced apoptosis of cultured rat hepatocytes

In former studies we have demonstrated that the incubation of cultured rat hepatocytes at 4°C leads to an increased cellular "chelatable iron pool" (i.e. cellular iron ions accessible to iron chelators) which later mediates the opening of the mitochondrial permeability transition pore eventually triggering apoptotic cell death [25,28,39]. This injurious mechanism to hepatocytes is likely to play a role under the conditions of liver transplantation.

**Figure 4:**
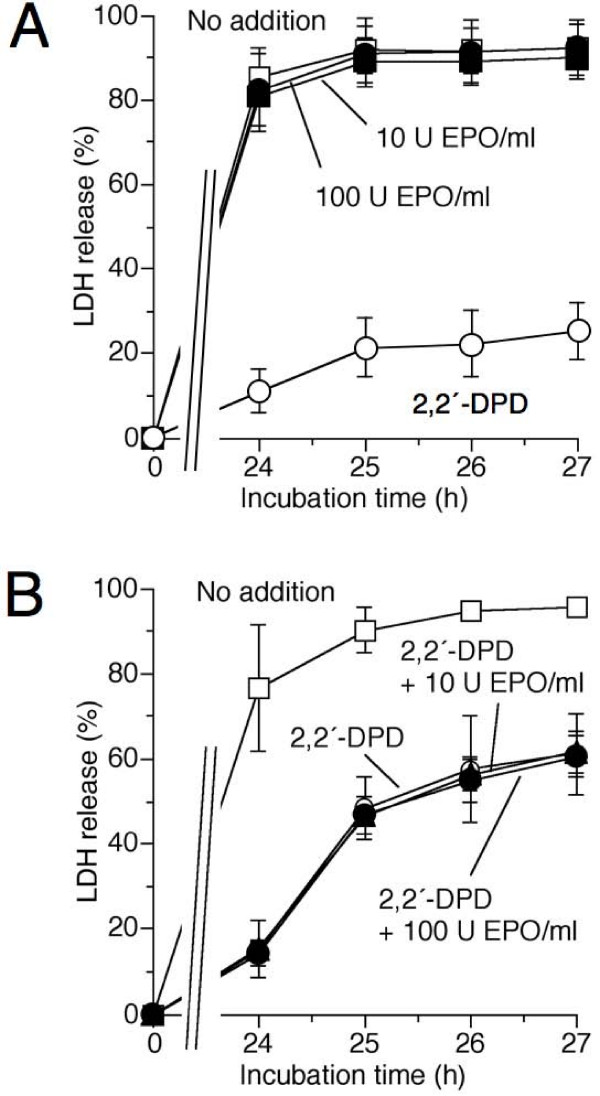
**Effect of rHu-EPO on cold-induced apoptosis of cultured rat hepatocytes**. (A) rat hepatocytes were pre-incubated in the presence or absence of rHu-EPO (10 or 100 U/ml) in L-15 medium for 18 hours at 37°C (74% N_2_/21% O_2_/5% CO_2_). Cells were then exposed to hypothermia (4°C) in University of Wisconsin solution with or without rHu-EPO. To prevent iron-dependent cold-induced apoptosis, the iron chelator 2,2'-dipyridyl (2,2'-DPD, 100 μM) was added prior to cold incubation. In (B), the effect of rHu-EPO on the iron-*in*dependent component of cold-induced apoptosis is shown. Cells were pre-incubated with rHu-EPO for 18 hours and then exposed to hypothermia in L-15 medium with or without rHu-EPO and/or 2,2'-DPD (100 μM). The occurrence of cell injury (including late apoptosis) was assessed by the release of lactate dehydrogenase (LDH). Values represent means ± S.D. of 3 independent experiments. (A) Open squares: hypothermia/rewarming, no addition; closed circles: hypothermia/rewarming, 100 U rHu-EPO/ml; closed squares: hypothermia/rewarming, 10 U rHu-EPO/ml; open circles: hypothermia/rewarming, 100 μM 2,2'-DPD. (B) Open squares: hypothermia/rewarming, no addition; open circles: hypothermia/rewarming, 100 μM 2,2'-DPD; closed circles: hypothermia/rewarming, 100 μM 2,2'-DPD and 10 U rHu-EPO/ml; closed triangles: hypothermia/rewarming, 100 μM 2,2'-DPD and 100 U rHu-EPO/ml.

Initially, experiments were performed in University of Wisconsin solution, as cold incubation in this solution is known to result in hepatocyte cold-induced injury that solely depends on the iron-dependent mechanism described above [25]. When cultured hepatocytes were incubated in University of Wisconsin solution at 4°C, about 90% of the cells died within 24 hours cold incubation/3 hours rewarming (Figure 4A). The bidentate iron chelator 2,2'-dipyridyl, forming a redox-inactive complex with cellular chelatable iron, almost completely prevented loss of cell viability. In contrast, rHu-EPO (2, 10, 50 or 100 U/ml; 2 or 18 hours pre-incubation) had no effect on the course of cell death (Figure 4A; data shown for 10 and 100 U rHu-EPO/ml and 18 hours pre-incubation). As to be expected from these results, rHu-EPO had also no protective effect on cell injury induced by redox-active labile iron ions (15 μM Fe(III)-bis(8-hydroxyquinoline); data not shown, n = 3), known to induce a mitochondrial permeability transition and subsequent apoptotic cell death in hepatocytes [40].

As cultured rat hepatocytes are known to die from both an iron-dependent (see above) and a weaker iron-*in*dependent pathway when the cells are incubated in cold L-15 medium [27,28], we studied whether rHu-EPO prevents the iron-*in*dependent component of cold-induced cell injury. The iron chelator 2,2'-dipyridyl strongly decreased loss of cell viability (Figure 4B). RHu-EPO, however, did not decrease the remaining iron-*in*dependent component of the cold-induced injury.

### Detection of EPOR mRNA in perfused rat livers and cultured rat hepatocytes. Effects of rHu-EPO

**Figure 5:**
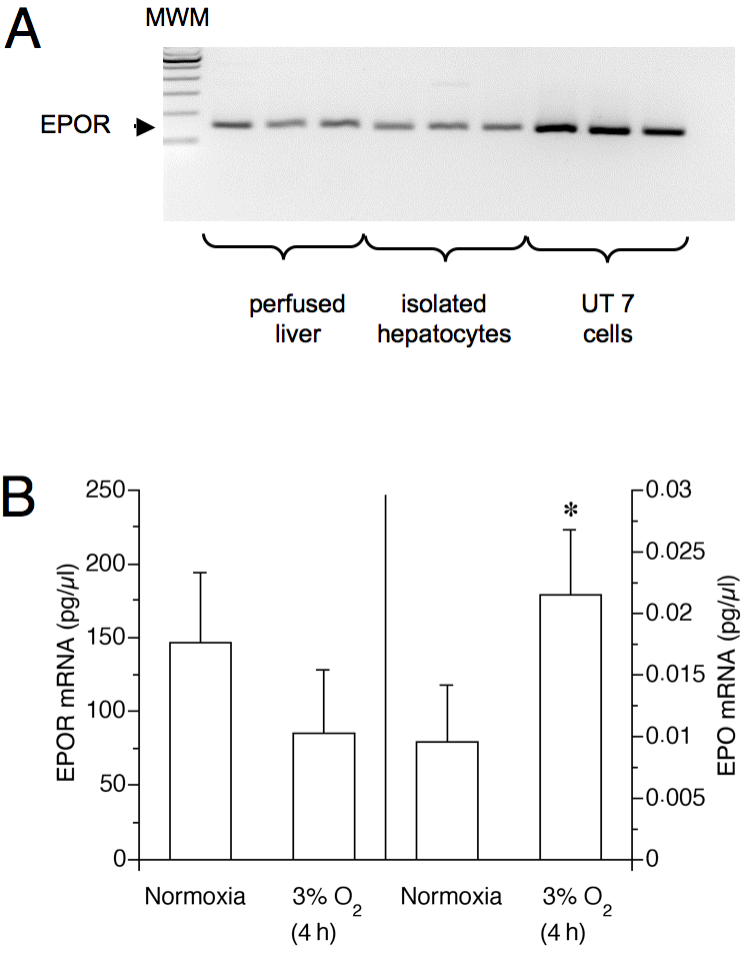
**Oxygen dependence of hepatocyte mRNA expression of EPOR and EPO**. (A) qualitative PCR of EPOR cDNA reverse transcribed from mRNA. Subsequent to their isolation hepatocytes were cultured in L-15 medium (37°C) for 20–22 hours at normoxia (74% N_2_/21% O_2_/5% CO_2_). Total RNA was extracted from the hepatocytes or from fresh tissue of perfused livers. Using primer EPOR1 and EPOR2 (see Methods), a single PCR product (arrow) was obtained which was sequenced and proved to be from the EPOR. For comparison, data from UT-7 cells ate shown. Levels of EPOR mRNA in liver tissue were around 152 ± 27 pg/μl RNA. (B) Real time PCR quantitation of EPOR and EPO mRNA. Hepatocytes were incubated for further 4 hours under normoxia or hypoxia (3% O_2_). Afterwards total RNA was extracted as in (A). 5 to 6 samples per treatment were analysed. Shown are mean values + S.D. *p < 0.05 vs. respective incubations at normoxia; MWM: molecular weight marker.

Primers designed to span the whole cDNA derived from the transcript revealed full length EPOR transcripts in cultured hepatocytes and perfused livers. In addition, primers spanning the membrane proximal and membrane spanning part of the EPOR confirmed expression of EPOR mRNA in all our samples from livers and hepatocytes (Figure 5). Qualitative RT-PCR revealed a single amplification product of hepatocyte and liver EPOR mRNA (Figure 5A) which was identical in size with our positive EPOR control (UT-7 cells). Levels of EPOR expression did not change during culture of hepatocytes when compared with freshly isolated liver tissue but were lower than in UT-7 cells (Figure 5A). When the mRNA was quantified by real time RT-PCR we found that hypoxia (mild hypoxia, 3% O_2_) decreased EPOR mRNA of hepatocytes to about 60% of the values under normoxia within 4 hours of incubation (Figure 5B). Since quantitative real time RT-PCR revealed a more than two-fold increase of EPO mRNA under hypoxia as compared to normoxic controls a general decrease of gene expression does not account for the lower EPOR mRNA levels under hypoxia. The addition of rHu-EPO (10 or 100 U/ml; 2 or 18 hours pre-incubation) did not affect EPOR mRNA levels under normoxic and moderate hypoxic conditions (4, 12 or 24 hours at 3% O_2_; data not shown; n = 3 – 4).

To verify the presence of the EPOR protein, Western blot analysis was carried out. Unfortunately, however, a specific antibody to EPOR is not available at present. Using a polyclonal antibody for the EPOR, we detected several bands of about the predicted size but could not reliably identify the EPOR protein, a results which is in full agreement with Elliott et al. [41] who questioned the specificity of the EPOR antibodies used. All bands around the predicted size of EPOR showed no change in their intensity upon treatment with rHu-EPO (10 or 100 U/ml; 2 or 18 hours pre-incubation; data not shown; n = 3).

### Activation of EPOR signalling in UT-7 cells but not in hepatocytes and perfused livers

**Figure 6:**
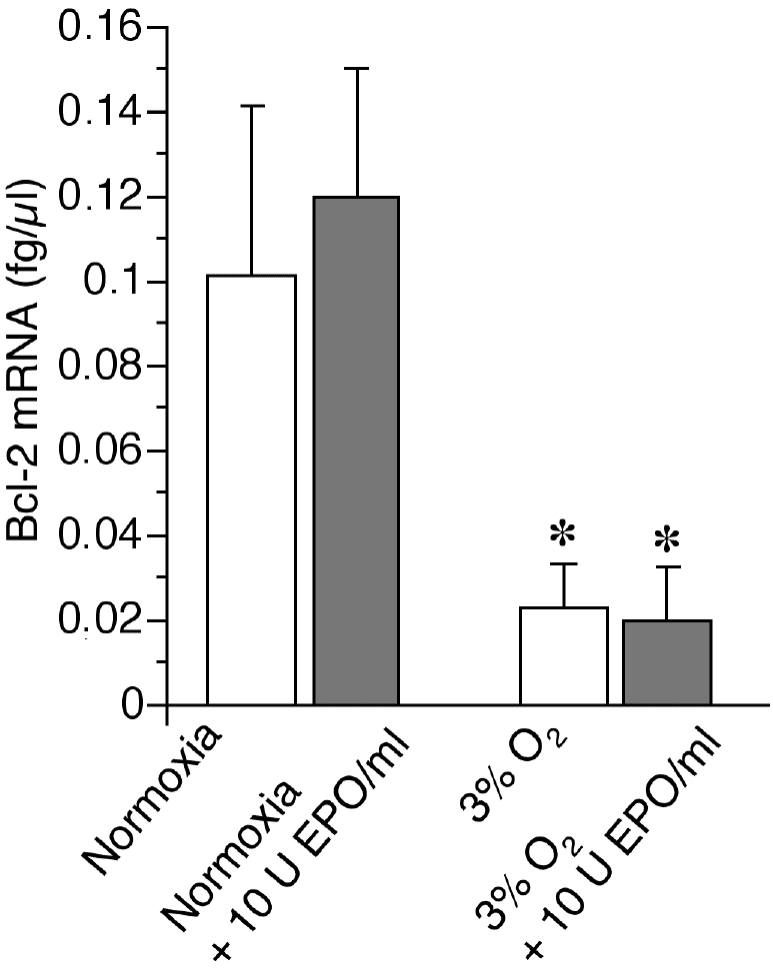
**Effect of rHu-EPO on Bcl-2 expression**. Quantitative (RT-)PCR from total RNA was performed as described in Methods after a 30 hours incubation (18 h preincubation, 12 h incubation) of hepatocytes with or without rHu-EPO (10 U/ml) under normoxia or hypoxia (3% O_2_). Data obtained for hepatocytes from 6 separate animals are shown. Data represents means + S.D. *p < 0.05 vs. respective incubations at normoxia.

The mRNA of the classical EPOR downstream target, the anti-apoptotic Bcl-2 gene, was detected in cultured hepatocytes. However, Bcl-2 mRNA was not induced by rHu-EPO treatment (10 U/ml for 30 h; Figure 6). In fact, Bcl-2 mRNA strongly decreased under hypoxia and rHu-EPO was not able to overcome this effect. Even much higher doses of EPO added for 24 h had no effect on Bcl-2 expression (Table 1).

**Table 1:** Effects of high rHu-EPO concentrations on Bcl-2 mRNA levels in rat hepatocytes

Treatment	Normoxia	Hypoxia (3% O_2_)
Control	0.11 ± 0.03 fg/μl	0.05 ± 0.02 fg/μl
EPO 50 U/ml	0.09 ± 0.03 fg/μl	0.04 ± 0.01 fg/μl
EPO 100 U/ml	0.08 ± 0.03 fg/μl	0.04 ± 0.02 fg/μl

NOTE. Quantitative (RT-)PCR from total RNA was performed as described in Methods after a 24 hours incubation of cultured rat hepatocytes with or without high doses of rHu-EPO under normoxia or hypoxia (3% O_2_). Data are the means ± SD of 4 separate experiments with hepatocytes different animals each.

**Figure 7:**
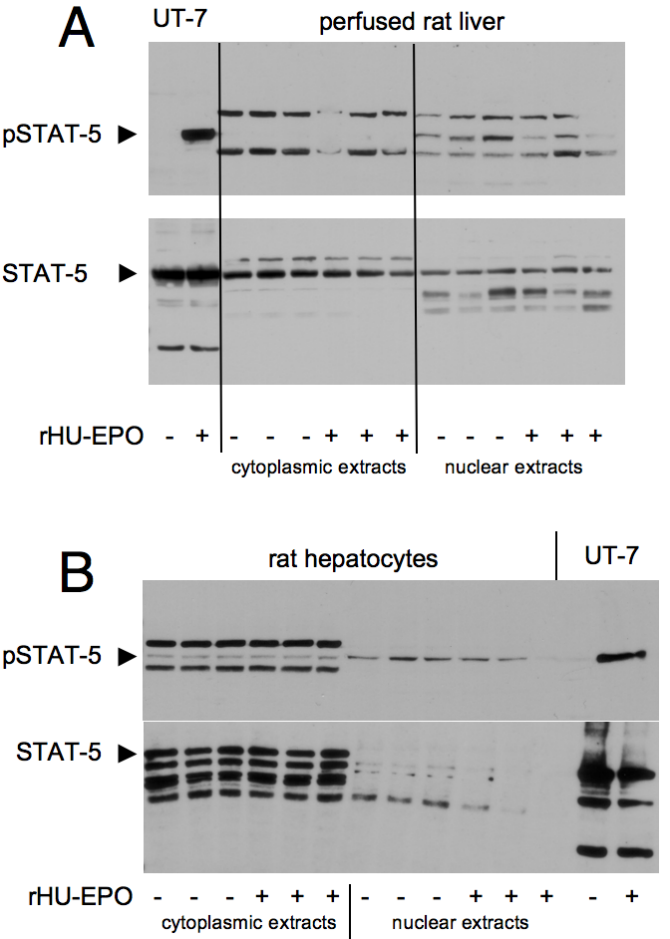
**Effect of rHu-EPO on the phosphorylation of STAT-5 in rat livers and hepatocytes**. Rat livers were perfused with 8.9 U rHU-EPO/ml and cultured hepatocytes incubated with 10 U/ml (both for 15 minutes). Afterwards, cytoplasmic and nuclear extracts were subjected to Western blotting. As a positive control for EPOR signalling whole cell extracts of the rHu-EPO-responsive cell line UT-7 were used that were treated with (or depleted from) rHU-EPO (10 U/ml for 15 minutes). In (A) total STAT-5 and pSTAT-5 were determined for liver extracts. In (B) corresponding results are shown for rat hepatocytes. The blots represent results of 6 experiments with different animals.

**Figure 8:**
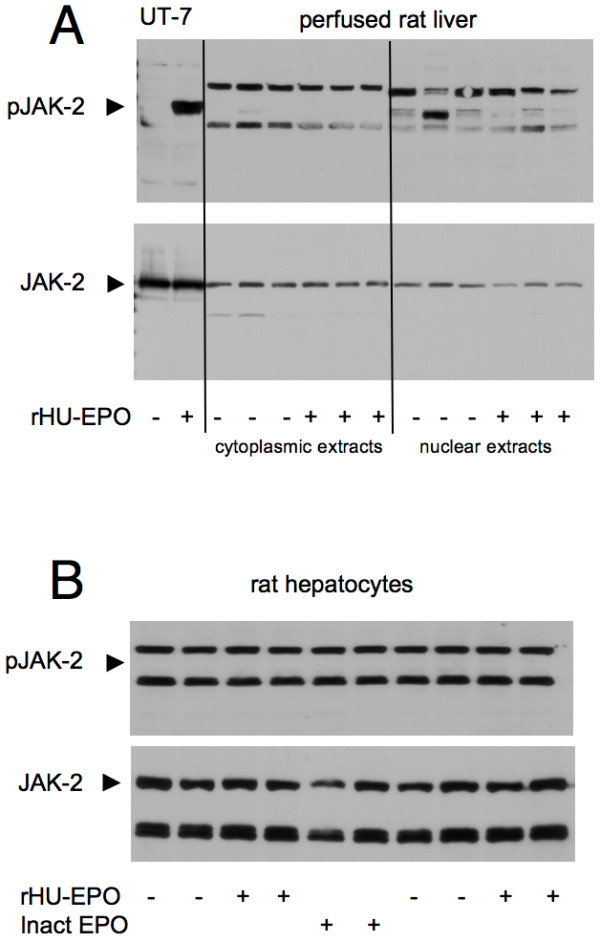
**Effect of rHu-EPO on the phosphorylation of JAK-2 in rat livers and hepatocytes**. Rat livers were perfused with 8.9 U rHU-EPO/ml and cultured hepatocytes incubated with 10 U/ml (both for 15 minutes). Afterwards, cytoplasmic and nuclear extracts were subjected to Western blotting. As a positive control for EPOR signalling whole cell extracts of the rHu-EPO-responsive cell line UT-7 were used that were treated with (or depleted from) rHU-EPO (10 U/ml for 15 minutes). Total JAK-2 and pJAK-2 were determined for extracts from (A) of perfused liver or (B) total cellular extracts from cultured hepatocytes. The blots represent results of 6 experiments with different animals.

To more directly study EPOR-induced intracellular signalling, EPO-responsive activation of JAK-2, STAT-3 and STAT-5 was determined in perfused livers and cultured hepatocytes. Rat livers were perfused *in situ *with KH buffer with or without rHu-EPO (8.9 U/ml) for 15 minutes. In general, cytoplasmic extracts contained more STAT-5 protein than nuclear extracts (Figure 7A). Nuclear extracts showed moderate phosphorylation of STAT-5 which was not affected by EPO. Cultured hepatocytes showed equal levels of STAT-5 protein but also no increase in phosphorylated STAT-5 after treatment with rHu-EPO (10 U/ml for 15 minutes (Figure 7B) or 18 hours; data not shown). Similar results were obtained for JAK-2: non-phosphorylated JAK-2 was found in livers and hepatocytes irrespective of EPO treatment and changes in phosphorylation of JAK-2 were not observed (Figure 8). Neither STAT-3 nor pSTAT-3 levels in hepatocytes and the livers were altered by rHu-EPO (data not shown).

For both STAT-5 and JAK-2, phosphorylation in UT-7 cells served as positive controls for the activation of classical EPOR downstream signalling: Withdrawal of rHu-EPO for 1 hour strongly reduced the phosphorylation of STAT-5 (Figure 7A and 7B) and JAK-2 (Figure 8A) while the signalling components were strongly phosphorylated upon treatment with rHu-EPO (10 U/ml for 15 minutes).

## Discussion

Hypoxic cell injury, hypoxia-reoxygenation injury and cold-induced apoptosis to cultured hepatocytes are in vitro models of injurious processes occurring to the hepatocyte in vivo during warm or cold ischemia and upon subsequent warm reperfusion of the liver. They cover a wide range of injurious mechanisms from necrotic to apoptotic cell injury [34,35]. Independent of the injurious model used, however, rHu-EPO was without any protective effect in these in vitro models. Thus, there is a discrepancy between the in vitro results obtained under standardized conditions here and the known protection provided by rHu-EPO against ischemia-reperfusion injury to the liver in vivo [4-9].

We did not find any evidence for EPO-dependent signalling either in the cultured hepatocytes or in the liver perfused in situ. RHu-EPO was not capable of activating JAK-2, STAT-3 and STAT-5, i.e. decisive components of EPO-mediated signal transduction, or of inducing Bcl-2, a well-known effector molecule of EPO-mediated protection. Only the measurements focusing directly on EPOR provided somewhat ambiguous results; although they as well do not suggest the presence of a functional EPOR. On the one hand, EPOR mRNA was detectable in the rat hepatocytes and perfused livers in line with data obtained from the liver of neonatal pigs [42] but in contrast to a previous study on mouse liver where no EPOR-specific PCR product was detectable anymore after birth [43]. Interestingly, while this manuscript was prepared Pinto et al. reported EPOR activation in mouse hepatocytes which was blocked by an anti-EPOR antibody [44]. Unfortunately, the authors of that study performed no experiments to define postreceptor signalling which makes a direct comparison with this study difficult but it remains to be resolved whether EPO preparations activate other signalling pathways than the classical JAK-2/STAT-5 cascade.

In our cells, EPOR mRNA expression was not stimulated by exogenous EPO which contrasts to the behaviour of other cell types such as erythroid progenitor cells, vascular endothelial and neuronal cells [12,45-47]. EPOR protein could unfortunately not be demonstrated, due to the unavailability of a specific antibody. However, clear evidence for EPOR signal transduction pathways in the rat liver has not been presented yet. Even for the in vivo experiments on ischemia-reperfusion injury to the liver and on liver regeneration following partial liver resection, where protection by rHu-EPO has been reported, no significant downstream activation of STAT-3 or VEGF mRNA expression has been documented [8,10].

Absence of protective EPO signalling fully explains the absence of protection by rHu-EPO in the cultured hepatocytes. This, however, raises the question about the mechanism of the liver protection by rHu-EPO in vivo and thus about the underlying reason for the apparent discrepancy between our in vitro and the published in vivo results. Decisive pathophysiological mechanisms contributing to the in vivo ischemia/reperfusion injury to the liver are mediated or enhanced by endothelial and Kupffer cells, i.e. by liver cells other than hepatocytes [35,48]. RHu-EPO may directly or indirectly act on these cells and thus influence the injurious response. Further, systemic effects of rHu-EPO on the cardiovascular, respiratory and neuronal system may modulate the outcome of ischemia/reperfusion injury to the liver in vivo. Other known indirect effects of rHu-EPO that may play a role are the suppression of the inflammatory response via down-regulation of NF-κ and AP-1 [49] and promotion of liver regeneration via stimulation of haematopoietic cells located in the liver [10]. In line with this, Yilmaz et al. [4] reported that „It may be that EPO acts upon the liver via mechanisms other than the tyrosine kinase pathway, i.e. that the protective effects of EPO on liver and nerves operate via different mechanisms."

Another or additional explanation for the in vivo liver protection by commercial rHu-EPO preparations is the high glycine concentration of the stock solutions. Unfortunately, no controls with heat-inactivated rHu-EPO have been performed in the in vivo studies. Assuming that a stock solution containing 10,000 U rHu-EPO and 5 mg glycine/ml was used, the application of 1,000–5,000 U/kg body weight, as performed in vivo, would increase the glycine blood concentration from around 180 μM [50] to 280–690 μM; given a blood volume of 65 ml/kg rat and provided that there is no glycine uptake by tissue (which of course does occur). Even higher glycine concentrations would be reached when stock solutions containing less rHu-EPO were used since their glycine content is always maintained at 5 mg/ml. Which stock solutions have been used is not clearly given in the in vivo studies cited above. Nevertheless, already ≥ 66 μM glycine significantly protected isolated hepatocytes form hypoxia/reoxygenation injury in the present study and slightly more (100 μM) in a previous one [36].

## Conclusion

In conclusion, we demonstrated that rHu-EPO has no protective effect on hypoxic injury, hypoxia-reoxygenation injury and cold-induced apoptosis to isolated cultured rat hepatocytes. This is in line with the absence of a protective EPO-dependent signalling in these cells as well as in the rat liver perfused in situ with rHu-EPO. We suggest that the protection provided by rHu-EPO in vivo against ischemia-reperfusion and other causes of liver injury does not result from a direct effect on hepatocytes.

## Competing interests

The authors declare that they have no competing interests.

## Authors' contributions

TB carried out and evaluated most of the cell viability studies regarding the effects of hypoxia-reoxygenation and Fe(III)/8-hydroxyquinoline and was involved in drafting the manuscript. PF determined and evaluated activated JAK-2, STAT-3 and STAT-5 and quantified EPOR, EPO and Bcl-2 mRNA. JF designed the experiments regarding the immunoassays and real-time PCRs and participated in drafting the manuscript. UR designed and coordinated the experiments regarding hypothermic injury/cold-induced apoptosis. KP carried out and evaluated some cell viability studies regarding the effects of hypoxia-reoxygenation. JE participated in the design of the study and critically discussed the clinical aspects. SF coordinated the experiments regarding the immunoassays and real-time PCRs. HdG conceived of the study and participated in its design and the drafting of the manuscript. FP performed the in situ perfusions of rat livers, participated in the concept/coordination of the study and in the drafting of the manuscript. All authors read and approved the final manuscript.

## Pre-publication history

The pre-publication history for this paper can be accessed here:

http://www.biomedcentral.com/1471-230X/9/26/prepub
